# P-229. Epidemiology of Catheter-Related Bloodstream Infections in Italian Intensive Care Units: Data from the Italian Network for the Evaluation of ICU Interventions

**DOI:** 10.1093/ofid/ofae631.433

**Published:** 2025-01-29

**Authors:** Camilla Genovese, Matilde Perego, Giovanni Scaglione, Emanuele Palomba, Giulia Giordano, Valentina Breschi, Giuseppe De Nicolao, Simone Milanesi, Bruno Viaggi, Andrea Gori, Stefano Finazzi, Marta Colaneri

**Affiliations:** Infectious Diseases and Immunopathology, Department of Clinical Sciences, Università di Milano, Luigi Sacco Hospital, Milan, Italy, Milano, Lombardia, Italy; Laboratory of Clinical Data Science, Department of Public Health, Istituto di Ricerche Farmacologiche Mario Negri IRCCS, Ranica, BG, Italy, Ranica, Lombardia, Italy; Division of Infectious Diseases, Luigi Sacco Hospital, University of Milan, Milan, Italy, Milano, Lombardia, Italy; Infectious Diseases and Immunopathology, Department of Clinical Sciences, Università di Milano, Luigi Sacco Hospital, Milan, Italy, Milano, Lombardia, Italy; Department of Industrial Engineering, University of Trento, Trento, Italy, Trento, Trentino-Alto Adige, Italy; Department of Electrical Engineering, Eindhoven University of Technology, 5600 MB Eindhoven, Netherlands, Eindhoven, Noord-Brabant, Netherlands; Department of Mathematics, University of Pavia, Pavia, Italy, Pavia, Lombardia, Italy; Department of Mathematics, University of Pavia, Pavia, Italy, Pavia, Lombardia, Italy; Anesthesia and Intensive Care, Careggi University Hospital, Florence, Italy, Firenze, Toscana, Italy; Infectious Diseases and Immunopathology, Department of Clinical Sciences, Università di Milano, Luigi Sacco Hospital, Milan, Italy, Milano, Lombardia, Italy; Laboratory of Clinical Data Science, Department of Public Health, Istituto di Ricerche Farmacologiche Mario Negri IRCCS, Ranica, BG, Italy, Ranica, Lombardia, Italy; Department of Biomedical and Clinical Sciences, University of Milan, Milan, Italy, Milano, Lombardia, Italy

## Abstract

**Background:**

Intensive care unit (ICU) acquired bloodstream infections (BSIs) are an important cause of morbidity and mortality. The use of invasive devices, such as endovascular catheters is associated with greater risk of developing such infections. Our study focuses on the epidemiology of ICU-acquired catheter-related BSIs (CR-BSIs) in Italy from 2014 to 2022.

**Table 1.**
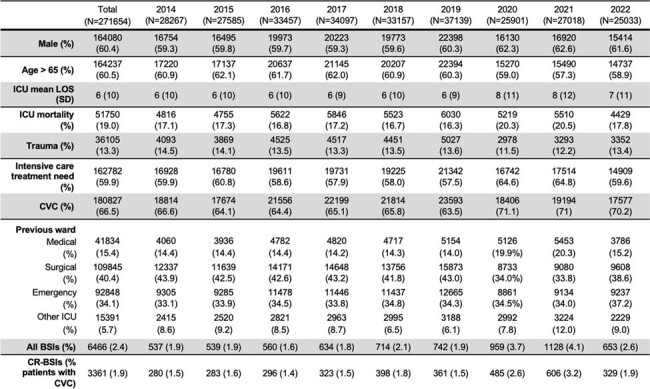
Characteristics of ICU admitted patients from 2014 to 2022. LOS, length of stay; SD, standard deviation; CVC, central venous catheter; BSI, bloodstream infection; CR-BSI, catheter-related bloodstream infection.

With intensive care treatment need we mean the number and percentage of patients who were admitted to ICU for intensive care treatment, therefore excluding those admitted for post-surgical monitoring or invasive procedure execution.

**Methods:**

We retrieved data from the PROSAFE project, a prospective, observational, multicentric study involving 135 Italian ICUs. Clinical data were continuously collected by physicians from January 2014 to December 2022. CR-BSIs episodes were defined in accordance with the National Healthcare Safety Network. Multi-drug resistant Gram-negative bacteria (MDR-GNB) were defined as resistant to at least one carbapenem.

Continuous variables were summarized with mean and standard deviation, while categorical data were presented as counts and percentages.

**Figure 1.**
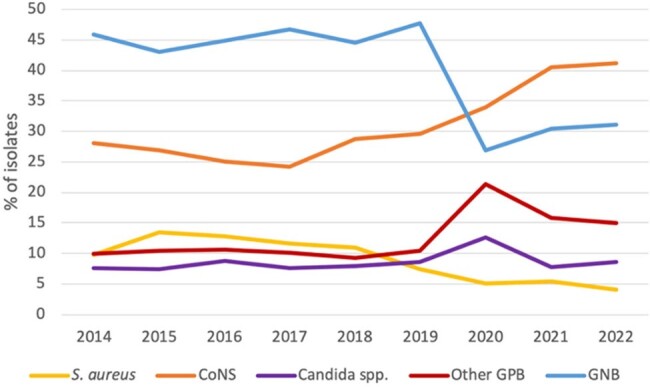
Percentage of species isolated from blood cultures of patients with ICU-acquired catheter-related bloodstream infection from 2014 to 2022. S. aureus, Staphylococcus aureus; CoNS, Coagulase negative Staphylococci; GPB, Gram positive bacteria, GNB; Gram negative bacteria. Other GPB include Streptococci and Enterococci.

**Results:**

A total of 271654 patients were included in our study (Table 1). The number of episodes of CR-BSIs was 3361. The in-ICU mortality of patients with CR-BSIs was 28.5%. The incidence of CR-BSIs slightly increased through the years (1.1% in 2014 vs 1.9% in 2022), peaking in 2020-2021. Regarding the microbiology, most bacteria were Gram positive (GP), representing around half of the involved pathogens (Figure 1). CR-BSIs due to coagulase-negative staphylococci (CoNS) increased over the years (from 28.9% in 2014 to 41.2% in 2022), particularly since the COVID-19 pandemic.

More than a third of CR-BSIs were caused by GNB, 22.7% of which were MDR. The most common pathogen was *Klebsiella pneumoniae*, which represented 13.4% of all CR-BSIs. An overall decrease of MDR-GNB was noted (Figure 2).

CR-fungemias represented almost 9% of all CR-BSIs, with a peak at 12% in 2020.

**Figure 2.**
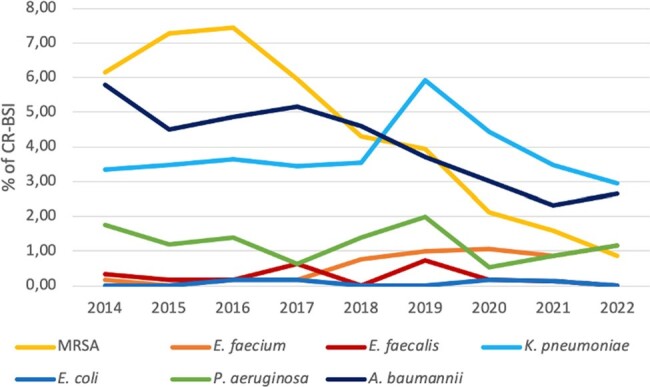
Percentage of resistant pathogens isolated from blood cultures of patients with ICU-acquired catheter-related bloodstream infection from 2014 to 2022.

Resistant pathogens were defined as resistant to methicillin for Staphylococcus aureus, resistant to vancomycin for Enterococci and resistant to at least one carbapenem for Gram-negative rods. CR-BSI, catheter-related bloodstream infection; MRSA, methicillin-resistant Staphylococcus aureus, E. faecium, Enterococcus faecium; E. faecalis, Enterococcus faecalis; K. pneumoniae, Klebsiella pneumoniae; E. coli, Escherichia coli; P. aeruginosa, Pseudomonas aeruginosa; A. baumannii, Acinetobacter baumannii.

**Conclusion:**

Our study highlights the significant burden of CR-BSIs in Italian ICUs. Despite efforts to mitigate these infections, the incidence of CR-BSIs has shown a concerning upward trend, particularly driven by the rise in CoNS infections, notably since the onset of the COVID-19 pandemic. These findings underscore the ongoing challenge of combating CR-BSIs and emphasize the importance of continued surveillance and targeted interventions to mitigate their impact on patient outcomes in ICUs.

**Disclosures:**

**All Authors**: No reported disclosures

